# Expandable Gastroretentive Films Based on Anthocyanin-Rich Rice Starch for Improved Ferulic Acid Delivery

**DOI:** 10.3390/polym17172301

**Published:** 2025-08-25

**Authors:** Nattawipa Matchimabura, Jiramate Poolsiri, Nataporn Phadungvitvatthana, Rachanida Praparatana, Ousanee Issarachot, Ruedeekorn Wiwattanapatapee

**Affiliations:** 1 Department of Pharmaceutical Technology, Faculty of Pharmaceutical Sciences, Prince of Songkla University, Songkhla 90112, Thailand; nattawipa.nm@gmail.com (N.M.); 6210710004@email.psu.ac.th (J.P.); 6210710040@email.psu.ac.th (N.P.); 2 Faculty of Medical Technology, Prince of Songkla University, Songkhla 90112, Thailand; rachanida.p@psu.ac.th; 3 Department of Pharmacy Technician, Faculty of Public Health and Allied Health Sciences, Sirindhorn College of Public Health Trang, Praboromrajchanok Institute, 89 M.2 Kantang District, Trang 92110, Thailand; ousaneemu@gmail.com; 4 Phytomedicine and Pharmaceutical Biotechnology Excellence Center, Faculty of Pharmaceutical Sciences, Prince of Songkla University, Songkhla 90112, Thailand

**Keywords:** ferulic acid, expandable film, rice starch, chitosan, gastroretentive carrier

## Abstract

Ferulic acid (FA) is a bioactive compound known for its potent antioxidant and anti-inflammatory properties; however, its poor water solubility significantly limits its bioavailability and therapeutic potential. In this study, a solid dispersion of FA (FA-SD) was developed using Eudragit^®^ EPO via the solvent evaporation method, achieving a 24-fold increase in solubility (42.7 mg/mL) at a 1:3 drug-to-polymer ratio. Expandable gastroretentive films were subsequently formulated using starches from Hom-Nil rice, glutinous rice, and white rice, combined with chitosan as the primary film-forming agents, via the solvent casting technique. Hydroxypropyl methylcellulose (HPMC) K100 LV was incorporated as an adjuvant to achieve controlled release. At optimal concentrations (3% *w*/*w* starch, 2% *w*/*w* chitosan, and 2% *w*/*w* HPMC), the films exhibited favorable mechanical properties, swelling capacity, and unfolding behavior. Sustained release of FA over 8 h was achieved in formulations containing HPMC with either Hom-Nil or glutinous rice starch. Among the tested formulations (R6, G6, and H6), those incorporating Hom-Nil rice starch demonstrated the most significant antioxidant (10.38 ± 0.23 μg/mL) and anti-inflammatory (9.26 ± 0.14 μg/mL) effects in murine macrophage cell line (RAW 264.7), surpassing the activities of both free FA and FA-SD. These results highlight the potential of anthocyanin-rich pigmented rice starch-based expandable films as effective gastroretentive systems for enhanced FA delivery.

## 1. Introduction

Ferulic acid (4-hydroxy-3-methoxycinnamic acid, FA) is a naturally occurring phenolic compound with the molecular formula C_10_H_10_O_4_, found in various plants [1]. It is well known for its potent antioxidant [2,3] and anti-inflammatory properties [4,5,6]. In recent years, FA has been reported to possess therapeutic potential in the treatment and prevention of gastric ulcers. The therapeutic effects of FA are largely attributed to its ability to scavenge free radicals and enhance the activity of antioxidant enzymes such as superoxide dismutase and catalase [2,3]. By reducing oxidative stress, a key factor linked to various chronic diseases, FA may play a protective role in maintaining gastric and overall health [7]. Recent research has shown that FA exhibits gastroprotective effects against indomethacin-induced stomach ulcers in rats by reducing both oxidative stress and inflammation, two key factors in the development of gastric ulcers. Its gastroprotective effects are attributed to the suppression of lipid peroxidation, inhibition of neutrophil infiltration, and modulation of the antioxidant defense mechanisms [8]. FA’s anti-inflammatory activity is primarily mediated through the inhibition of pro-inflammatory cytokines such as tumor necrosis factor-α (TNF-α), interleukin (IL)-1β, and IL-6 [4,5]. Additionally, FA suppresses the activation of nuclear factor kappa B (NF-κB), a central regulator of the inflammatory response [6,9]. Given these properties, FA represents a promising candidate for the development of innovative therapeutic approaches for the treatment of gastric ulcers.

Despite its therapeutic potential, the clinical application of FA is hindered by its poor water solubility and short half-life, both of which significantly limit its bioavailability [10]. To overcome these limitations, several strategies have been developed to improve its solubility and extend its retention time in the stomach, ultimately enhancing absorption and therapeutic effectiveness. Solid dispersion (SD) technology is a widely studied strategy for enhancing the solubility of poorly water-soluble drugs by improving their wettability and converting the drug substance from a crystalline to an amorphous form [11]. The core principle of SD systems involves dispersing the poorly soluble drug within hydrophilic carriers to facilitate dissolution. Among these carriers, Eudragit^®^ EPO a functional copolymer composed of dimethylaminoethyl methacrylate, methyl methacrylate, and butyl methacrylate has gained significant attention for its role in pharmaceutical applications [12]. It has been successfully used to enhance the solubility of several active pharmaceutical ingredients, including ellagic acid [13], curcumin [14,15], glycosides [12], and rosuvastatin calcium [16].

The gastroretentive drug delivery system (GRDDS) is an innovative approach designed to achieve controlled and prolonged drug release. By extending the residence time of a drug in the stomach, this system enables site-specific delivery and supports both local and systemic therapeutic effects [17]. Among various GRDDS platforms, oral expandable films have gained attention due to their ability to increase in size beyond the diameter of the pyloric sphincter, thereby preventing premature passage into the intestine and allowing the drug to be retained in the stomach for an extended period [18]. Starch is one of the most commonly used materials for film production due to its low cost, non-toxic nature, and biodegradability. However, the application of starch-based films is limited by their poor mechanical properties, high water solubility, and brittleness [19]. To overcome these drawbacks, starch is often combined with other materials, especially with chitosan, to enhance film strength and performance. The expandable film has been widely applied in the development of formulations containing active ingredients intended for prolonging gastric retention, stomach-specific delivery and the treatment of gastric diseases. The film carriers are typically composed of a film-forming agent and a plasticizer.

According to earlier findings, Starch-chitosan showed promising characteristics for a gastroretentive expandable film. A variety of starches, including potato, mung bean, banana, rice, and corn starch, have been used to combine with chitosan [15,20,21,22]. Curcumin served as a model drug that was successfully incorporated into expandable film carriers. However, the results found that the physicochemical properties of the films were influenced by both the type and concentration of starch used [15]. Rice starch, glutinous rice starch and pregelatinized starch were also used to blend with chitosan as a carrier of resveratrol, ginger and quercetin [20,21,22]. The release profiles indicated that pregelatinized starch is a suitable formulation for prolonging the release of resveratrol, but not for curcumin. In another study, ginger-loaded glutinous rice starch expandable films exhibited good tensile strength and significant expansion in simulated gastric fluid. The different ratio of amylose and amylopectin was related to the properties of expandability in terms of water absorption, thermal behavior, and gelation [21].

Recently, there has been increasing interest in the use of pigmented rice in both food and pharmaceutical products. Hom-Nil, a variety of Thai black rice, is particularly noteworthy due to its high anthocyanin content, which is associated with various health benefits [23]. Anthocyanins in Hom-Nil rice exhibit significant anti-inflammatory and antioxidative effects [24]. This makes it an intriguing candidate for exploration as a material for film production, potentially enhancing the activity of active compounds. While previous studies have explored various starch types such as potato starch, corn starch, tapioca starch, rice starch, banana starch, glutinous starch, pre-gelatinize starch and mung bean starch [15,20,21,22], the use of pigmented, anthocyanin-rich rice starch as a functional excipient has not yet been fully explored. Moreover, the fact that different types of starch affect the drug release of each compound differently presents a valuable opportunity for further development as a delivery system for FA.

This study introduces a novel approach by utilizing pigmented Hom Nil rice starch as a multifunctional excipient in oral expandable films as a potential antioxidant carrier for effective FA delivery. To our knowledge, this is the first report exploring anthocyanin-rich rice starch in combination with chitosan for gastroretentive drug delivery of active compound. FA solubility was enhanced using solid dispersion technology with Eudragit^®^ EPO as the matrix. The most suitable FA-SD was used to prepare expandable films with different starches (Hom-Nil rice, glutinous rice, and white rice starch) and chitosan as film-former agent. Glycerin was included to enhance flexibility. Moreover, HPMC K100 LV was incorporated to offer sustained release delivery due to its gel-forming. FA-loaded films were characterized by weight, thickness, tensile strength, expansion, swelling, and release. Antioxidant activity was assessed using the DPPH assay, while anti-inflammatory effects were evaluated in RAW264.7 murine macrophage cells to confirm the potential for treating gastric diseases.

## 2. Materials and Methods

### 2.1. Materials

Ferulic acid (>98%, purity) was sourced from Chanjao Longevity Co., Ltd. (Bangkok, Thailand). Glycerin was purchased from P.C. Drug Center Co., Ltd. (Bangkok, Thailand). Eudragit^®^ EPO (average MW 150 kDa) was provided as a gift sample by Evonik Industries AG (Darmstadt, Germany). HPMC K100 LV (80–120 cp, 2% in water at 20 °C) was received from Colorcon Asia Pacific Pte Ltd. (Singapore). Shrimp shell chitosan with a molecular weight of 300–500 kDa and 85% degree of deacetylation was procured from BIO21 Thailand Co., Ltd. (Chonburi, Thailand). Hom-Nil black rice starch was from Save Life Products Co., Ltd. (Chiang rai, Thailand). Glutinous rice starch and white rice starch were purchased from Thai Flour Industry Co., Ltd. (Bangkok, Thailand). RAW264.7 cells (murine macrophage cell line) purchased from the American Type Culture Collection, ATCC (Manassas, VA, USA). Lipopolysaccharide (LPS, from *Escherichia coli*), indomethacin, Griess reagent and 2,2-diphenyl-1-picrylhydrazyl (DPPH) were purchased from Sigma Aldrich (St. Louis, MO, USA). Butylated hydroxytoluene (BHT) was obtained from Sigma Aldrich (Buchs, Switzerland) and ascorbic acid was provided by Chemsupply (Adelaide, Australia). Fetal bovine serum (FBS), Roswell Park Memorial Institute 1640 medium (RPMI-1640) medium, 3-(4,5-dime-thyl-2-thiazolyl)-2,5-diphenyl-2H-tetrazolium bromide (MTT), phosphate-buffer saline (PBS), trypsin EDTA 0.25%, trypan blue solution and penicillin-streptomycin (Pen-strep) were provided by Gibco (Invitrogen, Carlsbad, CA, USA). Dimethyl sulfoxide (DMSO) was sourced from Amresco (Solon, OH, USA). All other chemicals were of analytical or pharmaceutical grades.

### 2.2. Preparation of Ferulic Acid-Solid Dispersions

Ferulic acid-solid dispersions (FA-SDs) were prepared by solvent evaporation [14]. Eudragit^®^ EPO, as a hydrophilic polymer, was used as a carrier. Briefly, FA and the polymer were dissolved in ethanol at drug-to-polymer ratios of 1:1 to 1:4 to obtain a clear solution, and the solvent was removed by rotary evaporation at 40 °C (Hei-VAP Core, Heidolph Instruments GmbH, Schwabach, Germany). The dry solids (FA-SDs) were taken and pulverized using a mortar and pestle, followed by sieving to obtain 50–250 μm. Physical mixtures of FA with Eudragit^®^ EPO (FA-PMs) were prepared by dry mixing in the same ratios as the FA-SDs using a mortar, followed by sieving to obtain a uniform particle size. The resulting powders were protected from light and kept in air-tight containers at 25 °C in a desiccator.

### 2.3. Solubility of FA-SDs

The solubility of pure FA, FA-SDs and FA-PMs was evaluated using the flask shaking method as previously described [14]. An excess amount of each sample was added to a vial containing 1 mL of 0.1 N hydrochloric acid (HCl, pH 1.2) and mixed using a vortex mixer (Vortex-Genie 2, 50 Hz model, Scientific Industries Inc., Bohemia, NY, USA) for 10 min. The vials were then placed in a shaking water bath (SW22 Julabo, Seelbach, Germany) at 37 ± 0.1 °C for 48 h. After incubation, the suspensions were centrifuged at 6000× *g* rpm for 15 min (Kubota 5922B/N, Kubota, Tokyo, Japan). The supernatant was collected, filtered through a 0.45 µm membrane filter, appropriately diluted, and analyzed spectrophotometrically at 320 nm using a UV spectrophotometer (UV-1900i, Shimadzu, Kyoto, Japan). Each test was performed in triplicate.

### 2.4. Powder X-Ray Diffraction (PXRD) Studies

The crystalline properties of pure FA, Eudragit^®^ EPO, FA-SDs and FA-PMs were analyzed using powder X-ray diffractometry (Empyrean, PANalytical, Almelo, The Netherlands). The PXRD patterns were traced at a voltage of 40 kV and a current of 30 mA. The samples were scanned over the angular (2θ) range of 5–90° with a step size of 0.026° and a speed of 70.125 s at each step.

### 2.5. Preparation of Expandable Films Loaded with FA-SD

**Figure 1:**
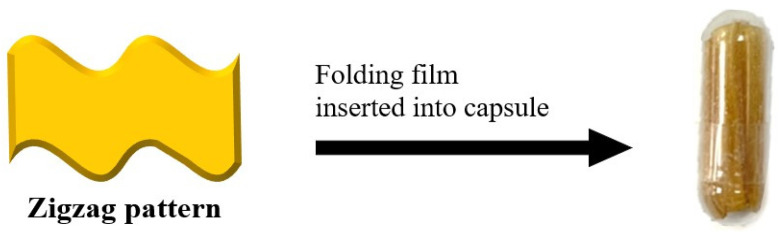
Schematic illustration of zigzag folding follow by insertion into a hard capsule.

Starch-based expandable films were prepared using the solvent casting method. The films were cast from a combination of three types of starch (white rice starch, glutinous rice starch, or Hom-Nil black rice starch) blended with chitosan. HPMC K100 and glycerin were used as a controlled-release polymer and plasticizer, respectively. The composition of FA-SD loaded expandable films is shown in Table 1. Chitosan, HPMC K100, and starch in varying proportions were introduced into 70 mL of 1% *v*/*v* acetic acid solution under continuous stirring. The mixture was then heated to 100 °C for one hour and subsequently cooled to room temperature while maintaining continuous stirring. Then, 2 g of FA-SD and glycerin were added to the matrix system, stirred until homogenous. The viscous solution was adjusted with 1% *v*/*v* acetic acid to obtain 100 g and poured into a glass Petri dish. After drying at 45 °C for 48 h, the polymeric films were meticulously detached and cut into rectangles of size 40 × 20 mm, followed by zigzag folding before insertion into a hard gelatin capsule size 00 (Figure 1). To prevent adhesion, the film samples were previously coated with microcrystalline cellulose.

### 2.6. Characterization of Expandable Films

#### 2.6.1. Measurement of Weight and Thickness

Ten rectangular samples (40 mm in length × 20 mm in width) from each formulation were weighed using a digital analytical balance (PRACTUM224-1S, Sartorius, Goettingen, Germany). The thickness of each sample was measured at five random points using a hand-held electronic digital vernier caliper (V6-154, Kovet Co., Ltd., Bangkok, Thailand). Results were reported as mean ± standard deviation.

#### 2.6.2. Drug Content

Rectangular film samples (40 mm in length × 20 mm in width) were soaked in 20 mL of 0.1 N hydrochloric acid (pH 1.2) for 4 h to dissolve chitosan. Methanol was then added to adjust the volume to 100 mL in a volumetric flask, and the solution was sonicated in an ultrasonic bath for 30 min to extract FA. Aliquots were diluted to a suitable concentration and filtered through a 0.45-μm membrane filter. The FA content was measured using a UV-Vis spectrophotometer (UV-1900i, Shimadzu, Kyoto, Japan).

#### 2.6.3. Tensile Strength

FA-loaded expandable films were cut into rectangular strips measuring 60 × 10 mm. Each strip was mounted between two clamps set 30 mm apart. To prevent film breakage at the clamp grooves, paper cards were placed between the clamps and the film surface. Six samples from each formulation were stretched at a crosshead speed of 2.0 mm/s until rupture. The applied force was recorded using Exponent 32 software. Tensile strength was calculated using Equation (1). Each sample was tested in quintuplicate, and the results were expressed as mean ± standard deviation.

Tensile strength (g/cm^2^) = force at break (g)/initial cross-sectional area of the sample (cm^2^) (1)

#### 2.6.4. Film Swelling Behavior

The swelling behavior of FA-SD-loaded expandable films was monitored over 8 h, as described by Kaewkroek et al. with slight modifications [21]. Rectangular film strips (10 × 20 mm) were initially weighed (W1) using a digital analytical balance (PRACTUM224-1S, Sartorius, Goettingen, Germany) and then immersed in 200 mL of 0.1 N hydrochloric acid (pH 1.2) maintained at 37 ± 0.5 °C. The sample weight was measured at predetermined time intervals (10, 20, 30, 60, 120, 240, 360, and 480 min) and recorded as W2. The percentage swelling index was calculated using Equation (2).

Swelling index (%) = (W2 − W1)/W1 × 100(2)

Each sample was repeated in triplicate and reported as mean ± standard deviation.

#### 2.6.5. Unfolding Behavior

The unfolding behavior of the expandable films was evaluated in 900 mL of 0.1 N hydrochloric acid (pH 1.2) using a USP 30 rotating basket dissolution apparatus (model PTWS 120D, Pharma Test, Hainburg, Germany). The rotation speed was set to 100 rpm. Film samples were cut to a size of 40 × 20 mm and folded in a zigzag pattern before being inserted into size 00 hard gelatin capsules. The capsules were placed in the dissolution medium, and the unfolding of the films was visually observed at time intervals of 10, 20, 30, 60, 120, 240, 360, and 480 min.

#### 2.6.6. Film Expansion

Capsules filled with zigzag-pattern expandable films were prepared as described in Section 2.6.5. The experiments were conducted in 200 mL of 0.1 N hydrochloric acid (pH 1.2), maintained at 37 ± 0.5 °C. After 8 h of immersion, the dimensions of the films were measured again. The expansion ratio was calculated by dividing the total area after full expansion by the initial area of the film. Each formulation was tested in triplicate, and results were reported as mean ± S.D.

Expanding ratio = initial area of the film (mm^2^)/total area of the film after 8 h (mm^2^) (3)

#### 2.6.7. In Vitro Release of FA from the Expandable Film

The release of FA was evaluated using a USP 30 rotating-basket dissolution apparatus (model PTWS 120D, Pharma Test, Hainburg, Germany). The test was conducted in 900 mL of 0.1 N hydrochloric acid (pH 1.2), maintained at 37 ± 0.5 °C, with a rotation speed of 100 rpm [21]. The test samples included FA-SD and selected FA-SD-loaded expandable films. For the film samples, zigzag-folded strips were inserted into hard gelatin capsules prior to testing. At predetermined time points (10, 20, 30, 60, 120, 240, 360, and 480 min), 5 mL of the dissolution medium was withdrawn and immediately replaced with an equal volume of fresh medium. FA content was quantified using a UV-Vis spectrophotometer at 320 nm. Each formulation was tested in triplicate, and results were expressed as mean ± S.D. Release profiles were plotted as cumulative percentage release versus time.

### 2.7. Determination of Antioxidant Activity

The antioxidant activity was evaluated using the 2,2-diphenyl-1-picrylhydrazyl (DPPH) radical scavenging assay, as described by [25]. Test samples including pure FA, FA-SD, FA-loaded expandable films (formulations R6, G6, and H6), and the corresponding blank films were dissolved and diluted in methanol to obtain final concentrations ranging from 1 to 200 µg/mL. Aliquots of 20 µL of each test sample were mixed with 180 µL of 0.1 mM DPPH methanolic solution in a 96-well plate. The plate was wrapped in aluminum foil and incubated in the dark at 25 °C for 30 min. After incubation, the absorbance of the residual DPPH was measured at 517 nm using a microplate reader (SPECTROstar Nano, BMG LABTECH, Ortenberg, Germany). Ascorbic acid and BHT were used as positive standards. The percentage inhibition of DPPH radicals was calculated using Equation (4).

Percentage inhibition = [Abs control − Abs sample/Abs control] × 100(4)

where Abs control is the absorbance of the control, and Abs sample is the absorbance of the sample.

### 2.8. Cytotoxicity Assay

The cell viability test was conducted using the MTT assay with RAW 264.7 cells (ATCC TIB-71, Manassas, VA, USA). RAW 264.7 cells were cultured in RPMI-1640 medium (Gibco^®^, Grand Island, NY, USA) supplemented with 10% fetal bovine serum (FBS, Gibco^®^, Grand Island, NY, USA) and 1% penicillin/streptomycin (Gibco^®^, Grand Island, NY, USA). Once confluence was reached, the cells were seeded into 96-well plates at a density of 1 × 10^5^ cells per well and incubated at 37 °C in a humidified atmosphere containing 5% CO_2_ for 24 h. The culture medium was then removed, and the wells were washed twice with phosphate-buffered saline (PBS). Samples—including pure FA, FA-SD, FA-loaded expandable films (formulations R6, G6, and H6), and their corresponding blank films were diluted to FA equivalent concentrations ranging from 6.25 to 80 μg/mL and added to the wells for incubation with the cells for 24 h.

Following treatment, the medium was removed and replaced with 0.5 mg/mL MTT solution. After a 3 h incubation, the supernatant was discarded and 200 µL of DMSO was added to dissolve the formazan crystals produced by metabolically active cells. Absorbance was measured at 570 nm using a microplate reader (Power Wave X, BioTek, Santa Clara, CA, USA). The percentage of cell viability was calculated using Equation (5).

% cell viability = [Abs sample/Abs control] × 100(5)

where Abs sample is the absorbance of the sample, and Abs control is the absorbance of the control (culture media). Each data point was repeated three times. Percentage cell viability was expressed as mean ± S.D.

### 2.9. Anti-Inflammatory Assay

The anti-inflammatory effect was assessed by measuring the inhibition of nitric oxide (NO) production, as previously described by [26]. The experiment was performed using RAW 264.7 macrophage cells, with lipopolysaccharide (LPS) employed as the inflammation inducer. RAW 264.7 cells were seeded into 96-well plates at a concentration of 1 × 10^5^ cells/mL and treated with either culture medium (control) or test samples, including pure FA, FA-SD, FA-SD-loaded expandable films (formulations R6, G6, and H6), and their corresponding blank films. The samples were applied at FA-equivalent concentrations ranging from 6.25 to 50 μg/mL, with or without 100 ng/mL of LPS. After 24 h of incubation, 100 μL of the culture supernatant was transferred to a new 96-well plate and mixed with 100 μL of Griess reagent at a 1:1 ratio. Nitrite concentration, as an indicator of NO production, was determined by measuring absorbance at 570 nm. The percentage inhibition of nitric oxide production was calculated using Equation (6):

% Inhibition = [1 – (Abs sample/Abs control)] × 100(6)

Abs control is the absorbance of cells-derived media exposed to LPS alone, whereas Abs sample is the absorbance of media in which cells were treated with the test sample and LPS. Indomethacin (NSAID) was applied as a positive control.

Results were repeated in triplicate and expressed as IC_50_ values by calculating as the concentration that inhibited NO production by 50%.

### 2.10. Statistical Analysis

All results were expressed as mean ± S.D. Statistical significance was evaluated using Student’s *t*-test and one-way analysis of variance (ANOVA), as appropriate. Differences between groups were considered statistically significant at *p* < 0.05.

## 3. Results and Discussion

### 3.1. Solubility of FA-SDs and FA-PMs

**Figure 2:**
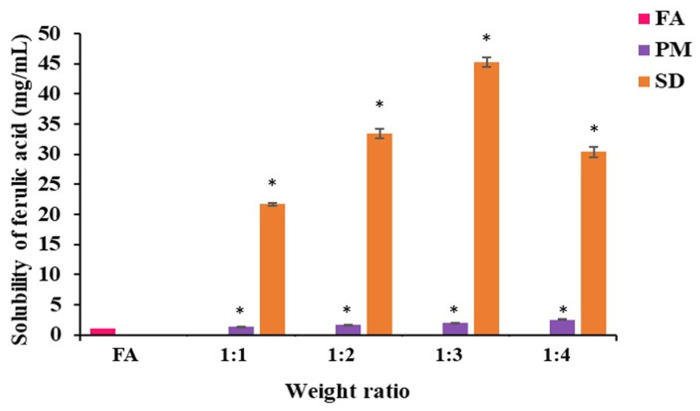
Solubility of pure FA, FA-solid dispersions (FA-SD), and FA-physical mixtures (FA-PM) in 0.1N HCl at 37 °C. * indicates a statistically significant difference (*p* < 0.05) compared with FA.

FA-SDs were prepared using the solvent evaporation method. After sieving, the final product was obtained as a cream-colored powder with a particle size ranging from 50 to 250 µm. The solubility of different ratios of FA-SDs, FA-PMs, and pure FA in 0.1 N HCl solution (pH 1.2) is shown in Figure 2.

Pure FA exhibited limited solubility in acidic medium, with a measured concentration of 1.77 mg/mL in 0.1 N HCl (pH 1.2). Notably, the solubility of FA-SDs prepared at drug to polymer ratios ranging from 1:1 to 1:4 was significantly enhanced compared to pure FA (*p* < 0.05). The highest solubility was observed at a drug to Eudragit^®^ EPO ratio of 1:3, reaching 45 mg/mL, corresponding to a 24-fold increase. However, a further increase in polymer content (1:4 ratio) resulted in a decline in solubility, likely due to steric hindrance that may impede effective drug–polymer interactions at higher polymer concentrations [27]. Similar behavior was reported for solid dispersion of tacrolimus [28], curcumin [14], and glycoside-rich centella extract with Eudragit^®^ EPO [12]. Moreover, a high content of hydrophilic polymer can increase viscosity, leading to the formation of a gel-like barrier that impedes drug diffusion and reduces the dissolution rate [29]. In the case of the physical mixtures, a modest increase in solubility was observed, approximately 1.9-fold compared to pure FA. Consequently, the FA–Eudragit^®^ EPO solid dispersion at a drug to polymer ratio of 1:3 was selected for further investigation.

### 3.2. Powder X-Ray Diffraction (PXRD Analysis)

**Figure 3:**
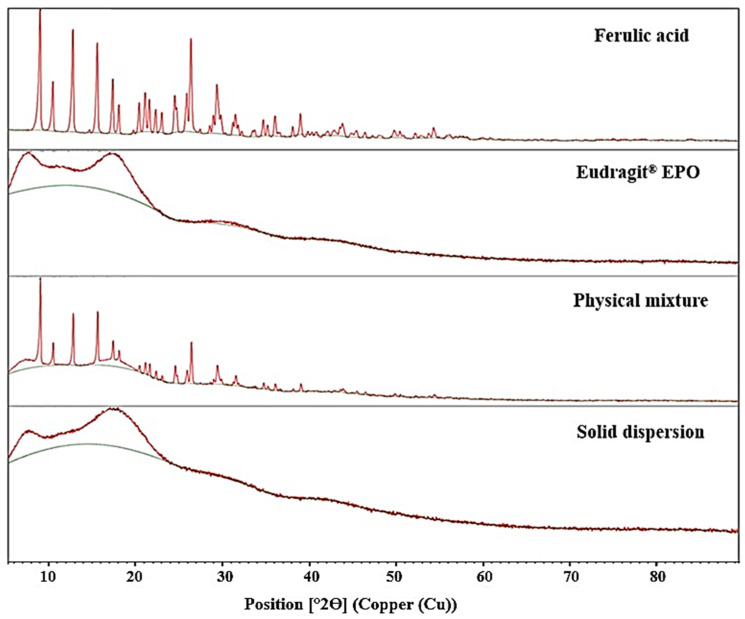
Powder X-ray diffraction spectra of pure FA, Eudragit^®^ EPO, FA-SD, and FA-PM.

The X-ray diffraction (XRD) patterns of pure FA, Eudragit^®^ EPO, FA-SD, and FA-PM are presented in Figure 3. Pure FA exhibited distinct and sharp diffraction peaks at 2θ values of 9.1°, 10.5°, 12.9°, 15.7°, 17.5°, and 26.5°, confirming its crystalline nature. In contrast, Eudragit^®^ EPO, employed as a hydrophilic carrier, displayed a broad halo without distinct peaks, indicative of its amorphous character. As amorphous materials generally possess higher free energy states than crystalline forms, this characteristic contributes to their enhanced solubility [30].

The XRD pattern of the FA-SD showed a marked reduction in the intensity of sharp crystalline peaks and the appearance of a broad halo, indicating the conversion of crystalline FA into an amorphous form. This transformation is consistent with solubility data, which demonstrate a significant enhancement in FA solubility upon solid dispersion formulation. These findings align with previous studies on solid dispersions, including ginger extract/PVP K30 [26], glycoside-rich *Centella asiatica* extract/Eudragit^®^ EPO [12], and quercetin/PVP K30 [31]. The diffractogram of FA-PMs retained the sharp peaks characteristic of pure FA, indicating that there was no significant interaction between FA and Eudragit^®^ EPO. This suggests that the physical blending in FA-PMs did not promote strong drug–polymer interactions.

### 3.3. Physicochemical Properties of FA-SD Loaded Expandable Films

#### 3.3.1. Weight and Thickness

**Figure 4:**
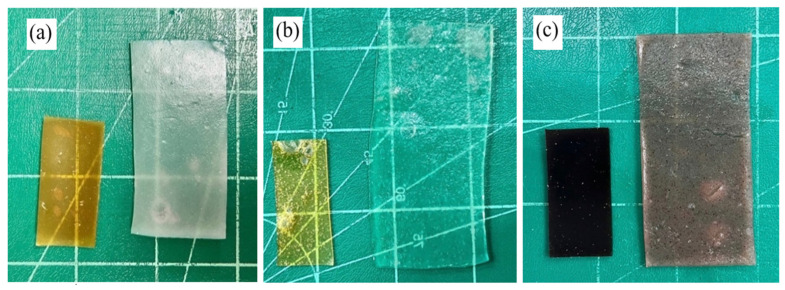
The appearance of the FA-SD loaded expansion films before and after the expansion test (**a**) R6, (**b**) G6, and (**c**) H6.

Expandable films composed of chitosan and various starches (white rice, glutinous rice, and Hom-Nil rice starch) were successfully prepared using the solvent casting method. Solvent casting is widely employed for film formation due to low cost and practicality [32]. All film formulations exhibited homogeneity, with varying colors. As shown in Figure 4, films prepared with white rice starch and glutinous rice starch were dark yellow and light yellow, respectively, while those made with Hom-Nil rice starch exhibited dark purple hues, likely due to the presence of anthocyanin compounds. The average weight of the expandable films ranged from 0.58 ± 0.01 g to 0.91 ± 0.02 g, as summarized in Table 2. The weight varied slightly depending on the concentrations of chitosan and HPMC. Similarly, the film thickness increased with the concentration of film components, ranging from 0.41 ± 0.01 mm to 0.94 ± 0.02 mm. The consistency in weight and thickness is evident from the small standard deviations.

#### 3.3.2. Tensile Strength of Films

Tensile strength was measured to assess the mechanical strength and flexibility of the films, as higher tensile strength reflects greater resistance to compressive forces encountered in the stomach during drug release. The results indicated that tensile strength was influenced by both the type of starch used and the concentration of chitosan in the formulation (Table 2). Films containing higher amounts of chitosan showed an increase in tensile strength (R3 > R2 > R1, G3 > G2 > G1, and H3 > H2 > H1) due to the formation of more intermolecular hydrogen bonds between chitosan and starch, which enhanced film strength [33]. When the amount of starches and chitosan was fixed, the tensile strength was also related to the increment of HPMC (R6 > R5 > R4, G6 > G5 > G4, and H6 > H5 > H4), as it formed intermolecular hydrogen bonds with chitosan [34]. Expandable films prepared with white rice starch exhibited the highest tensile strength, attributed to its high amylose content. Amylose has a linear molecular structure that contributes to increased film strength [35]. In contrast, films made from glutinous rice starch and Hom-Nil rice starch showed similar, lower tensile strength values, likely due to their lower amylose content. Furthermore, among the three starch types, white rice starch consistently produced stronger films than both glutinous and Hom-Nil rice starch [36,37].

#### 3.3.3. Expansion of Films

Film samples were folded in a zigzag manner and inserted into hard gelatin capsules (size 00). The expansion behavior of the films was evaluated in 0.1 N HCl solution (pH 1.2) over a period of 8 h. Upon exposure to the dissolution medium, all films demonstrated an increase in surface area, expanding approximately 2- to 5-fold, indicating their potential suitability for gastric retention applications (Table 2, Figure 4). The films made from Hom-Nil rice starch, particularly those with lower chitosan content (H1 and H2 formulations), exhibited disintegration during the experiment due to inadequate structural integration. Hence, the chitosan amount was fixed at 2% *w*/*w* before the addition of HPMC. As shown in Table 2, the incorporation of HPMC into the formulation enhanced the film’s hydrophilic properties, leading to greater expansion. This effect is attributed to the formation of pores within the film matrix, which facilitated increased fluid uptake [33]. At 2% *w*/*w* of HPMC, glutinous rice starch films (G6) exhibited the greatest expansion, increasing to 4.55 times compared with their original size, significantly larger than the expansion observed in white rice starch (R6) and Hom-Nil rice starch (H6). This enhanced expansion is directly correlated with the higher amylopectin content in glutinous rice starch, which promotes greater water absorption and swelling capacity [21]. Glutinous rice starch, being rich in amylopectin, demonstrated superior expansion compared to white rice starch and Hom-Nil rice starch. The incorporation of chitosan into the formulations tended to reduce the expansion of the films (G1 > G2 > G3). This phenomenon can be explained by the formation of intermolecular hydrogen bonds between the rice starch and chitosan, which reduced water penetration and consequently limited the swelling of the film [38,39].

#### 3.3.4. Swelling Behavior of Films

**Figure 5:**
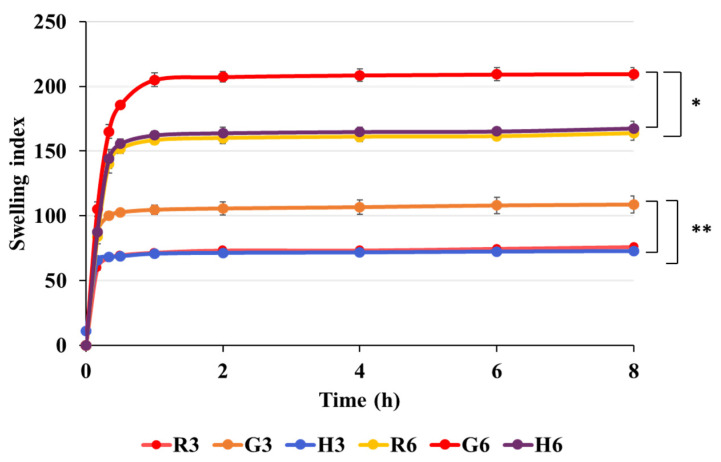
Swelling behavior of FA-SD loaded expandable film based on white rice, glutinous rice, and Hom-Nil rice starch with 2% *w*/*w* HPMC (Formulations R6, G6, and H6) or without HPMC (Formulations R3, G3, H3), the significant difference in swelling index were compared at 8 h. * Significant difference at *p* < 0.05 when comparing between G3 and R3 or H3, ** Significant difference at *p* < 0.05 when comparing between G6 and R6 or H6.

The water absorption capacity of the films was evaluated in 0.1 N HCl solution (pH 1.2) over an 8 h period, as shown in Figure 5. At a fixed starch concentration of 3% *w*/*w* and chitosan concentration of 2% *w*/*w*, films formulated with glutinous rice starch (G3) demonstrated significantly higher fluid absorption compared to those prepared with white rice or Hom-Nil rice starch (R3 and H3). The increased fluid absorption is likely due to the high amylopectin content in glutinous rice starch, indicating that starches rich in amylopectin exhibit superior water retention properties [21]. The ratio of amylose to amylopectin has been shown to play a critical role in modulating the swelling behavior of films and influencing the drug release rate [40]. Additionally, the film swelling was significantly pronounced in films containing HPMC due to the presence of more hydroxyl groups in the HPMC molecules [41]. Incorporation of HPMC, a hydrophilic polymer, significantly improved fluid uptake by inducing structural porosity in the films, leading to enhanced absorption rates compared to formulations without HPMC [33,42]. Similarly, the glutinous rice starch-based film (G6) exhibited greater swelling compared to the white rice starch (R6) and Hom-Nil rice starch (H6) films. In terms of time-dependent absorption behavior, the films exhibited rapid swelling within the first hour of immersion, after which the swelling index stabilized and remained constant over the 8 h period.

#### 3.3.5. Unfolding Behavior of Expandable Films

**Figure 6:**
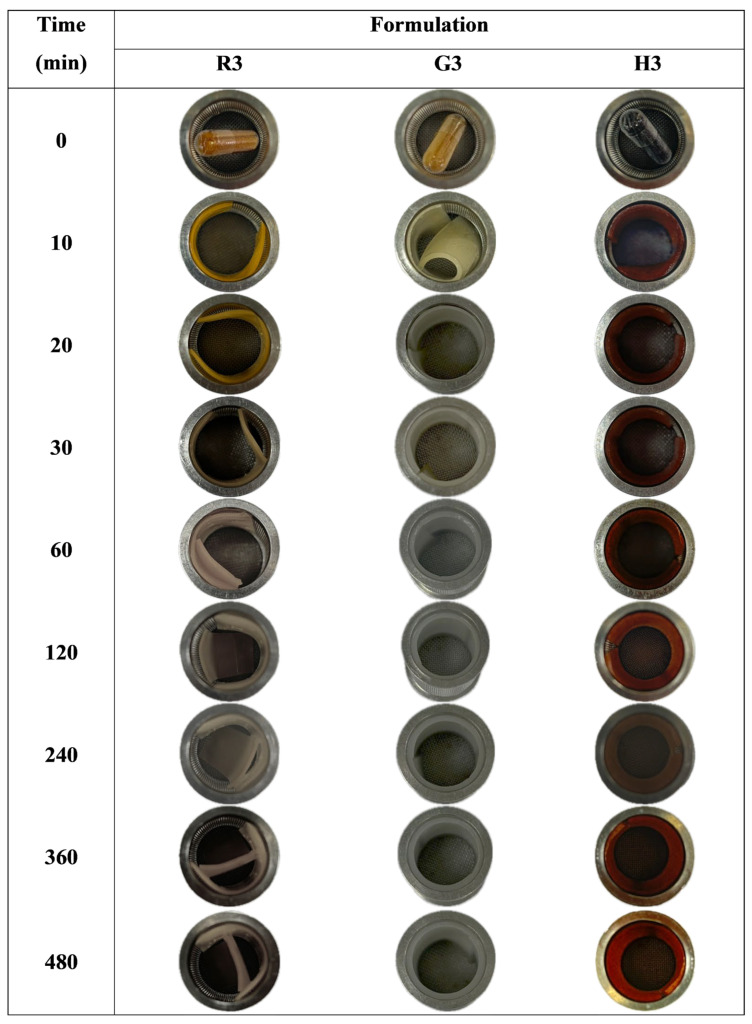
Unfolding behavior of FA-SD expandable films without HPMC: Effect of rice starch (R3), Glutinous rice starch (G3) and Hom-Nil rice starch (H3) at various time points in 0.1 N HCl solution, pH 1.2.

**Figure 7:**
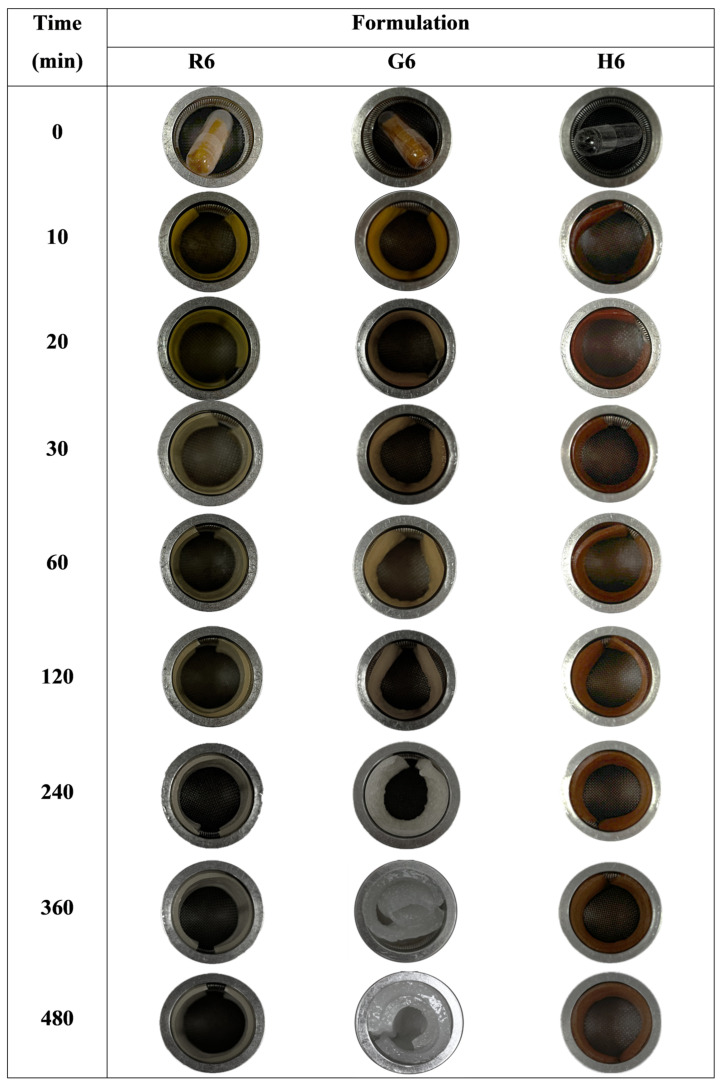
Unfolding behavior of FA-SD expandable films with HPMC Effect of rice starch (R3), Glutinous rice starch (G3) and Hom-Nil rice starch (H3) at various time points in 0.1 N HCl solution, pH 1.2.

The unfolding behavior of the expandable films was evaluated by inserting zigzag-folded films into size 00 hard gelatin capsules. The experiment was conducted in 0.1 N HCl solution (pH 1.2) over a period of 8 h. Test samples included films without HPMC (R3, G3, and H3) and films containing 2% *w*/*w* HPMC (R6, G6, and H6), as illustrated in Figure 6 and Figure 7. For the HPMC-containing formulations, the FA-SD expandable films gradually unfolded and reached full expansion within 10 min. The films maintained their rectangular shape throughout the test, demonstrating good structural integrity, except for formulation R3, which ruptured after 30 min (Figure 6). These results highlight the enhanced flexibility and elastic recovery of the films conferred by the inclusion of HPMC. As shown in Figure 7, the thickness of the unfolding films correlated with their swelling behavior, with glutinous rice starch films exhibiting greater swelling compared to those made with white rice or Hom-Nil rice starch.

#### 3.3.6. Release of FA from Films

**Figure 8:**
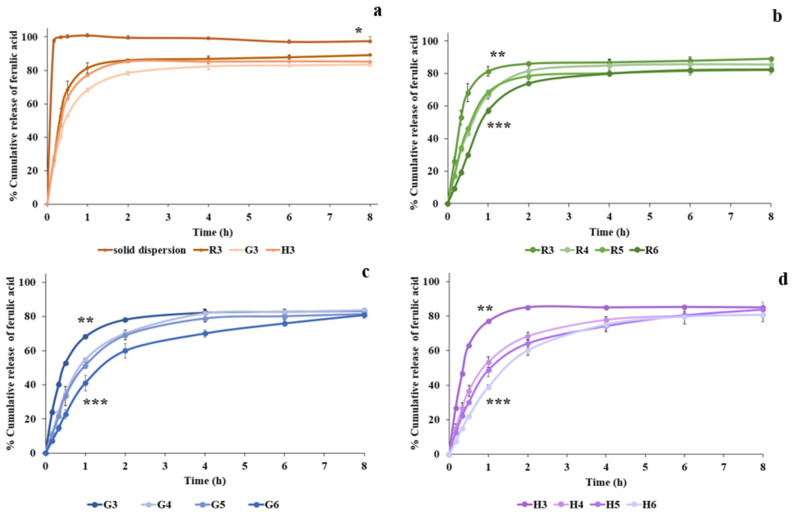
Release profile of FA from (**a**) FA-SD and non-HPMC (R3, G3 and H3) (**b**) White rice starch film with HPMC (**c**) Glutinous rice starch film with HPMC and (**d**) Hom-Nil rice starch film with HPMC in 0.1 N HCl (pH 1.2). Data reported as mean ± SD (*n* = 3). The significant differences in drug release were compared at 8 h. * Significant difference at *p* < 0.05 when comparing between % drug release of solid dispersion and the formulations. Figure 8b–d, the significant differences in drug release were compared at 1 h in order to compare between the retard release of the formulation without HPMC or with HPMC. ** Significant difference at *p* < 0.05 when comparing between % drug release of the formulation without HPMC and the formulations with HPMC. *** Significant difference at *p* < 0.05 when comparing the formulations containing HPMC.

The in vitro release of FA was conducted in 900 mL of 0.1 N HCl solution (pH 1.2) over an 8 h period. The FA-SD at a 1:3 drug to polymer ratio was selected for testing due to its significantly enhanced solubility. FA-SD expandable films, both with and without HPMC, were used to evaluate the release profile of FA, with starch and chitosan concentrations fixed at 3% *w*/*w* and 2% *w*/*w*, respectively. As shown in Figure 8, FA was rapidly released from the solid dispersion reaching nearly 100% release within 10 min. This result confirms that FA was successfully converted into an amorphous state, thereby significantly enhancing its dissolution.

Similarly to previous reports, the dissolution of various drugs such as curcumin, glycosides, quercetin, and rutin was enhanced by the solid dispersion process [12,15,31,43]. In the absence of HPMC, the FA-SD expandable film exhibited a rapid burst release within the first hour across all starch-based formulations (R3, H3, and G3), with release percentages as follows: white rice starch (81%), Hom-Nil starch (77%), and glutinous starch (68%). A plateau phase was observed after 2 h of exposure (Figure 8a). Hence, HPMC was incorporated into the selected formulations to retard the release. The effect of HPMC was further investigated by incorporating three concentrations, 0.5%, 1%, and 1.5% by weight, into the starch films (Figure 8b–d). These films exhibited a slow initial release, followed by a phase of gradual and sustained release over an 8 h period. White rice starch-based films exhibited an initially reduced FA release when combined with HPMC, particularly at 2% *w*/*w*. However, complete drug release was achieved within 2 h, although the total amount of FA released was slightly reduced. Glutinous rice starch and Hom-Nil rice starch films exhibited similar FA release profiles following HPMC incorporation. During the first hour, FA release was lower, approximately 40–50% *w*/*w* and was influenced by the HPMC content. An increase in polymer concentration raised the viscosity of the gel layer and extended the diffusional path length, resulting in a more pronounced delay in drug release [42]. Although HPMC delayed the initial release of FA, the total release eventually reached approximately 80% *w*/*w* in both glutinous rice starch and Hom-Nil rice starch films. Based on multiple properties including tensile strength, expandability, and FA release rate, rice starch-based films incorporating 2% *w*/*w* chitosan and 2% *w*/*w* HPMC were selected for in vitro cell studies.

#### 3.3.7. Antioxidant Activity

The 2,2-diphenyl-1-picrylhydrazyl (DPPH) radical scavenging assay is widely used to evaluate antioxidant activity due to its effectiveness in determining the radical scavenging ability of antioxidants [44,45]. The antioxidant activity of the samples was assessed based on their ability to scavenge DPPH radicals, as presented in Table 3. FA demonstrated strong antioxidant activity, with an IC_50_ value of 8.78 µg/mL, indicating 50% inhibition of DPPH free radicals. Its antioxidant mechanism has been associated with the electron-donating methoxy group at C-3 and hydroxyl group at C-4 on the benzene ring, which stabilize the phenoxy radical via resonance and thereby terminate free radical chain reactions [3]. Ascorbic acid and BHT, used as positive controls, exhibited IC_50_ values of 1.28 µg/mL and 11.77 µg/mL, respectively. Notably, FA-SD showed reduced antioxidant activity compared to pure FA, with a higher IC_50_ value of 14.01 µg/mL.

Film formulations containing FA as a solid dispersion (FA-SD), H6 (Hom-Nil rice starch), R6 (white rice starch), and G6 (glutinous rice starch), exhibited radical scavenging activity, with IC_50_ values of 10.38, 25.77, and 23.91 µg/mL, respectively. Among the film formulations, the Hom-Nil rice starch-based film (H6) exhibited significantly higher antioxidant activity. This can be attributed to the presence of anthocyanins in Hom-Nil rice, which are known for their antioxidant properties and their ability to form hydrogen bonds with radicals, thereby reducing free radicals [46]. These findings suggest that Hom-Nil rice starch could serve as an effective antioxidant carrier for FA delivery.

#### 3.3.8. Cytotoxicity

**Figure 9:**
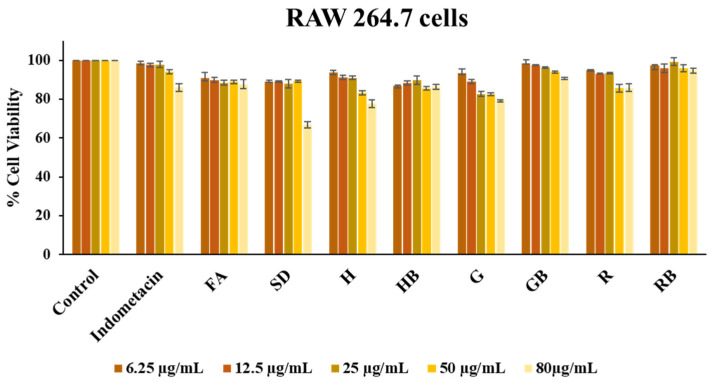
Cell viability of RAW 264.7 macrophages after 24h exposure to control (culture medium), ferulic acid (FA), solid dispersion (SD), FA-SD loaded expandable film based on white rice, glutinous rice, and Hom-Nil rice starch (Formulations H6, G6, and R6), and their blank formulations (HB, GB, and RB).

The cell viability of murine RAW 264.7 macrophages, used as a model for anti-inflammation, was assessed following exposure to pure FA, FA-SD, FA-SD loaded expandable films (H6, G6, and R6), and blank films, using the MTT assay. As shown in Figure 9, cell viability of RAW 264.7 cells remained above 80% across test concentrations ranging from 6.25 to 50 μg/mL, indicating non-toxicity. However, mild toxicity was observed at the higher concentration (80 μg/mL) for FA-SD.

Results expressed as a percentage of the control. Data show mean ± SD of triplicate determinations.

#### 3.3.9. Anti-Inflammatory Activity

The anti-inflammatory activity of pure FA, FA-SDs, FA-SD-loaded expandable films and their blank films was tested and compared with indomethacin. As summarized in Table 3, indomethacin, an NSAID used as a control, exhibited an IC_50_ value of 31.86 μg/mL. FA demonstrated a significant anti-inflammatory effect by inhibiting the production of pro-inflammatory cytokines, such as TNF-α, IL-6, and IL-1β [5]. FA-SD showed inhibitory activity against lipopolysaccharide (LPS)-induced NO production in RAW 264.7 cell line, with an IC_50_ value of 10.02 μg/mL, which was more potent than unformulated FA (28.90 μg/mL). This enhanced activity may be attributed to the FA-SD formulation with Eudragit^®^ EPO, which likely improves cellular uptake and anti-inflammatory efficacy. A similar result was previously reported for the enhanced cytotoxicity activity of a curcumin solid dispersion against the human gastric adenocarcinoma cell line [15]. The superior inhibitory activity was observed in formulation H6, which is composed of pigmented rice starch (Hom-Nil), compared to other rice starch films (G6 and R6), with IC_50_ values of 9.26, 14.05, and 12.86 μg/mL, respectively. These findings highlight the potential of Hom-Nil rice starch as a film carrier for stomach-specific delivery of FA-SD in the treatment of inflammatory diseases, such as gastric ulcers.

Based on these findings, it is suggested that incorporating the pigmented rice starch could enhance FA efficiency beyond individual effects. Although the precise mechanisms remain to be fully elucidated, it is possible that synergistic interactions occur between FA and anthocyanins through complementary antioxidant and anti-inflammatory pathways. FA exerts radical scavenging ability and can modulate inflammation by inhibiting pro-inflammatory cytokines such as TNF-α, IL-1β, and IL-6 [4,5] and enzymes such as cyclooxygenase (COX)-2 and nitric oxide synthase (iNOS) [47]. Additionally, FA suppresses the activation of NF-κB, a central regulator of the inflammatory response [6,9]. Anthocyanins, a class of flavonoids, also exhibit strong antioxidant activity due to the scavenging of free radicals, reactive oxygen species (ROS) and reactive nitrogen species [48]. It attenuates inflammation by downregulating pro-inflammatory mediators such as TNF-α, IL-1β, iNOS, monocyte chemoattractant protein-1, and COX-2, primarily via modulation of signaling pathways like NF-κB [48,49].

Recent studies suggest that combinations of other phenolic compounds can exert synergistic effects by enhancing antioxidant enzyme expression and suppressing ROS-mediated inflammatory responses [50]. These findings support the possibility that synergistic interactions between FA and anthocyanins may contribute to improved therapeutic efficacy in preventing or alleviating oxidative stress and inflammation-related conditions.

## 4. Conclusions

FA-SD-loaded expandable films were prepared using three different starches—white rice starch, glutinous rice starch, and Hom-Nil rice starch—combined with chitosan as a polymeric matrix, which helps prolong gastric residence time. The solubility of FA was primarily enhanced by its combination with Eudragit^®^ EPO in amorphous SD forms. The selected films completely unfolded in 0.1 N HCl solution (pH 1.2) after 10 min of immersion and maintained their structural integrity for 8 h. Incorporating HPMC into the expandable films slowed the initial release phase and sustained FA release (>80%). Compared to the other starch-based films, Hom-Nil rice starch demonstrated superior antioxidant activity and anti-inflammatory effects on RAW 264.7 macrophage cells by reducing NO production. This study highlights the promising potential of pigmented rice starch, particularly Hom-Nil rice starch, as an effective carrier for stomach-specific delivery of FA, offering a promising strategy for FA delivery in the stomach. In future studies, in vivo evaluation should be performed to confirm the gastric retention and pharmacological performance of the developed films in a biological system.

## Figures and Tables

**Table 1:** Compositions of FA-SD starch-based expandable films.

Formulation	Composition (% *w*/*w*)				
	Rice Starch	Glutinous Rice Starch	Hom-Nil Rice Starch	Chitosan	HPMC K100 LV
R1	3.0	-	-	1.0	-
R2				1.5	-
R3				2.0	-
R4	3.0	-	-	2.0	0.5
R5				2.0	1.0
R6				2.0	2.0
G1	-	3.0	-	1.0	-
G2				1.5	-
G3				2.0	-
G4	-	3.0	-	2.0	0.5
G5				2.0	1.0
G6				2.0	2.0
H1	-	-	3.0	1.0	-
H2				1.5	-
H3				2.0	-
H4	-	-	3.0	2.0	0.5
H5				2.0	1.0
H6				2.0	2.0

Both FA-SD and glycerin were fixed at 2% *w*/*w*.

**Table 2:** Physical properties of FA-SD-loaded expandable films.

Formulation	Weight (g)	Thickness (mm)	Film Expansion (fold)	Tensile Strength (g/cm^2^)
R1	0.58 ± 0.01	0.49 ± 0.05	2.80 ± 0.09	5.00 ± 0.19
R2	0.64 ± 0.01	0.52 ± 0.06	2.53 ± 0.23	9.67 ± 0.51
R3	0.68 ± 0.03	0.62 ± 0.03	breakage	15.33 ± 2.36 *^R1^
R4	0.72 ± 0.03	0.72 ± 0.04	1.98 ± 0.06	17.53 ± 0.57
R5	0.73 ± 0.02	0.80 ± 0.05	2.40 ± 0.11	20.12 ± 0.77
R6	0.73 ± 0.01	0.83 ± 0.03	2.45 ± 0.07 ***	26.57 ± 1.50 **^R4,R5^
G1	0.67 ± 0.01	0.41 ± 0.01	4.32± 0.57	3.70 ± 0.05
G2	0.70 ± 0.01	0.50 ± 0.09	3.91 ± 0.43	5.29 ± 0.39
G3	0.74 ± 0.06	0.63 ± 0.02	3.32 ± 0.65	12.95 ± 0.08 *^G1,G2^
G4	0.76 ± 0.06	0.73 ± 0.06	3.06 ± 0.02	13.52 ± 1.20
G5	0.78 ± 0.01	0.88 ± 0.08	3.52 ± 0.01	17.69 ± 0.61
G6	0.91 ± 0.02	0.94 ± 0.02	4.55 ± 0.16	24.22 ± 0.99 **^G4,G5^
H1	0.63 ± 0.06	0.62 ± 0.05	breakage	4.60 ± 0.08
H2	0.67 ± 0.06	0.63 ± 0.09	breakage	6.36 ± 0.68
H3	0.80 ± 0.08	0.66 ± 0.01	2.59 ± 0.12	8.38 ± 0.68 *^H1^
H4	0.82 ± 0.03	0.69 ± 0.03	2.72 ± 0.09	14.95 ± 0.24
H5	0.84 ± 0.03	0.73 ± 0.02	2.82 ± 1.34	18.60 ± 1.53
H6	0.86 ± 0.01	0.75 ± 0.02	2.89 ± 0.93 ***	23.78 ± 1.47 **^H4,H5^

When R = rice starch, G = glutinous rice starch and H = Hom-Nil rice starch. * Significant difference at *p* < 0.05 when compared within the same starch formulation without HPMC. ** Significant difference at *p* < 0.05 when compared within the same starch formulations with HPMC. *** Significant difference at *p* < 0.05 when compared with formulation G6.

**Table 3:** In vitro antioxidant and anti-inflammatory activity of FA-loaded expandable films.

Test Sample	Antioxidant Activity IC_50_ (µg/mL)	Anti-Inflammatory Activity IC_50_ (µg/mL)
Ferulic acid (FA)	8.78 ± 0.19	28.90 ± 0.29
Ferulic acid-solid dispersion (FA-SD)	14.01 ± 0.52	10.02 ± 0.05
Hom-Nil rice starch-based film (H6)	10.38 ± 0.23	9.26 ± 0.14
Blank Hom-Nil starch film (HB)	>100	>50
Glutinous rice starch-based film (G6)	23.91 ± 1.90	14.05 ± 0.88
Blank glutinous rice starch film (GB)	>100	>50
Rice starch-based film (R6)	25.77 ± 1.15	12.86 ± 0.04
Blank rice starch film (RB)	>100	>50
BHT	11.77 ± 0.50	-
Ascorbic acid	1.28 ± 0.34	-
Indomethacin	-	31.86 ± 0.42

Data represents mean ± S.D. of triplicate determinations (*n* = 3).

## Data Availability

The original contributions presented in this study are included in the article. Further inquiries can be directed to the corresponding author(s).
